# Stress-Related Immune Markers in Depression: Implications for Treatment

**DOI:** 10.1093/ijnp/pyw001

**Published:** 2016-01-16

**Authors:** Martina M. Hughes, Thomas J. Connor, Andrew Harkin

**Affiliations:** Neuroimmunology Research Group, Department of Physiology, School of Medicine & Trinity College Institute of Neuroscience (Drs Hughes and Connor), and Neuropsychopharmacology Research Group, School of Pharmacy and Pharmaceutical Sciences & Trinity College Institute of Neuroscience, Trinity College, Dublin, Ireland (Dr Harkin).

**Keywords:** stress, depression, immune, inflammation

## Abstract

Major depression is a serious psychiatric disorder; however, the precise biological basis of depression still remains elusive. A large body of evidence implicates a dysregulated endocrine and inflammatory response system in the pathogenesis of depression. Despite this, given the heterogeneity of depression, not all depressed patients exhibit dysregulation of the inflammatory and endocrine systems. Evidence suggests that inflammation is associated with depression in certain subgroups of patients and that those who have experienced stressful life events such as childhood trauma or bereavement may be at greater risk of developing depression. Consequently, prolonged exposure to stress is thought to be a key trigger for the onset of a depressive episode. This review assesses the relationship between stress and the immune system, with a particular interest in the mechanisms by which stress impacts immune function, and how altered immune functioning, in turn, may lead to a feed forward cascade of multiple systems dysregulation and the subsequent manifestation of depressive symptomology. The identification of stress-related immune markers and potential avenues for advances in therapeutic intervention is vital. Changes in specific biological markers may be used to characterize or differentiate depressive subtypes or specific symptoms and may predict treatment response, in turn facilitating a more effective, targeted, and fast-acting approach to treatment.

## Stress Response System

Stress has long been identified as a risk factor for major depression, while markers of immunological stimulation and inflammation frequently characterize subgroups of severely depressed patients. This review assesses the relationship between stress and the immune system, with a particular interest in the mechanisms by which stress impacts immune function, in an effort to identify stress-related immune markers and potential avenues for advances in therapeutic intervention.

An individual’s capacity to deal with stress is largely controlled by the hypothalamic pituitary adrenal (HPA) axis. A dysregulated stress response system, evidenced by hyperactivity of the HPA-axis, represents a vulnerability factor for major depressive disorders and is one of the most consistent findings in patients with major depression (Carroll et al., 1976a, 1976b; Nemeroff et al., 1984; Pariante and Lightman, 2008), although others suggest that atypical depression may be characterized by HPA axis hypoactivity (Gold and Chrousos, 2002; Parker et al., 2003).

Under normal physiological conditions, the HPA axis is self-regulatory and activity is curtailed via negative feedback inhibition; glucocorticoid receptor (GR) binding in the hypothalamus and the pituitary inhibits the activity of the HPA axis and the subsequent release of glucocorticoids from the adrenal cortex. Additionally, while cytokines such as interleukin-1beta (IL-1β) and IL-6 secreted by immune cells can directly act on the GR in the hypothalamus, activating the HPA axis, glucocorticoids also act to inhibit the synthesis and secretion of inflammatory cytokines, evidenced by the inhibition of endotoxin-induced fever in animals treated with exogenous glucocorticoids (Coelho et al., 1992), inhibition of inflammation, the production of IL-12 by antigen presenting cells, and suppression of proinflammatory cytokine expression (Elenkov and Chrousos, 2002; Pace and Miller, 2009).

## The Immune Response

In response to stress, injury or invading pathogens the body’s first line of defence is the activation of the nonspecific innate immune response. The primary task for the host’s innate immune cells is to detect the pathogen and mount an immediate defensive response, resulting in the activation of the inflammatory response, followed by the initiation of a highly diverse, antigen-specific, adaptive immune response and subsequent immunological memory (Hoffmann et al., 1999; Medzhitov, 2001). Upon encountering pathogen associated molecular pathogens or foreign antigen, monocytic innate immune cells respond by producing a variety of cytokines such as IL-1β, IL-6, and IL-12 along with tumor necrosis factor alpha (TNF-α).

**Figure 1.**
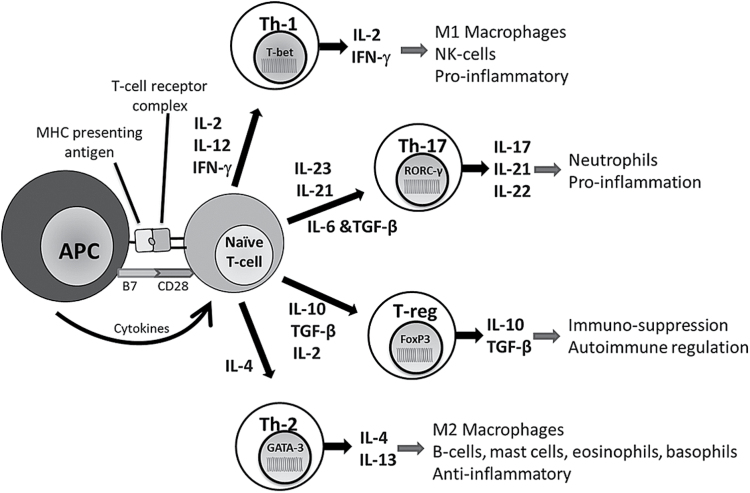
Simplified overview of T-helper (Th) cell subsets and their functions. The key effector molecules of the inflammatory response system (IRS) are the heterogeneous group of small cell signalling peptides, cytokines (Connor and Leonard, 1998). Released from numerous cell types in the brain and periphery, cytokines act in synergy or antagonistically to direct specific immune responses through the orchestration of immune cell trafficking and the cytokine-induced differentiation of immune cells with roles in innate, cytotoxic, cell-mediated, humoral, and autoimmunity in association with immune suppression (Borish and Steinke, 2003; Commins et al., 2010).

The subsequent process of dendritic cell maturation followed by antigen presentation, cytokine production, and co-stimulation from the innate response system results in the activation of lymphocytes, the key effector cells of the adaptive immune response (Hoebe et al., 2004). Depending on the nature of the signals received, distinct T-cell subsets are activated; proinflammatory T-helper (Th)-1 cells are induced in the presence of IL-2, IL-12, or interferon-gamma (IFN-γ) (Romagnani, 2000), while Il-17, IL-23, and a combination of Il-6 and transforming growth factor beta (TGF-β) are inducers of Th-17 cell activation (Bettelli et al., 2006). The antiinflammatory cytokine IL-4 mediates the differentiation of Th-2 cells (Romagnani, 2000), while the immuno-regulatory mediators IL-10 and TGF-β are potent inducers of T-reg cell differentiation (Bettelli et al., 2006) (Figure 1).

Under normal physiological conditions, proinflammatory Th-1 cells play a key role in protecting the host against intracellular and viral pathogens (Zhu et al., 2010). Alternatively, antiinflammatory Th-2 cells are necessary for the induction of humoral immunity, B cell activation, and the production of Immunoglobulin E antibody and immunoglobulin G neutralizing antibody, in addition to dampening the proinflammatory response (Romagnani, 2000). However, in disease states, it is thought that the balance between pro- and antiinflammatory mediators is lost in favor of a persistent proinflammatory phenotype.

Furthermore, Th-17 cells are thought to be involved in acute inflammatory responses, orchestrating the recruitment of neutrophils to epithelial cells and promoting growth and integrity of epithelial barriers (Wilson et al., 2007). On the other hand, T-reg cells function to maintain immunological homeostasis, protecting against auto-immunity and downregulating T-cell activation and inflammatory cytokine production via NFκB inhibition and/or the depletion or uptake of the lymphocyte-proliferation mediator, IL-2 (Thornton and Shevach, 1998; Bettelli et al., 2005, Popmihajlov and Smith, 2008; Cheng et al., 2011). However, dysregulation of the inflammatory response has detrimental consequences. Th-17 cells are widely implicated in numerous disorders such as irritable bowel syndrome and arthritis (Tesmer et al., 2008), while decreased T-reg cell suppressive function, as a consequence of inflammation, has been reported in rheumatoid arthritis (Nie et al., 2013).

Of particular interest in depressive disorders are the cytokines IL-β, IL-6, and TNF-α. IL-1β is produced by many immune cells, which include mononuclear phagocytes, neutrophils, endothelial cells, and microglia, the resident immune cells in the brain, in response to bacterial endotoxins and other pathogenic agents recognized by pathogen recognition receptors (Dinarello, 1988). The production of IL-1β has a key role to play in the induction and maintenance of the adaptive immune response, promoting increased expression of IL-2 receptors and IL-2 secretion, thereby impacting on T-cell differentiation and B cell activation (Gillis and Mizel, 1981; Kaye et al., 1984).

IL-6 is produced by many cells types such as T and B cells, endothelial cells, and hepatocytes, amongst others; however, the major producers of IL-6 are innate immune mononuclear phagocytes (Commins et al., 2010). IL-6 is a pleiotropic cytokine with both pro- and antiinflammatory effects. IL-6 is the strongest mediator of the acute phase response, stimulating the release of proteins such as C-reactive protein (CRP) and albumin from the liver along with playing a role in immune-mediated HPA axis activation, perhaps via direct interaction with IL-6 receptors in the paraventricular nucleus of the hypothalamus and thereby inducing the stress response (Lenczowski et al., 1999; Horn et al., 2000).

TNF-α is a multi-functional cytokine that plays a central role in mediating host defence against intracellular pathogens and bacterial endotoxin (Pfeffer, 2003). Similar to IL-6 and IL-1β, TNF is produced by many cell types, including neutrophils, lymphocytes, and endothelial cells; however, the major producers of TNF-α are mononuclear phagocytes (Beutler and Cerami, 1989). TNF-α plays a critical role in the inflammatory response, promoting inflammation via the stimulated expression of IL-1β and IL-6 in association with promoting lymphocyte proliferation.

## Stress as A Trigger for Activating the Immune System

Further to the findings reviewed by Connor and Leonard (1998), a wealth of evidence has emerged supporting the notion that inflammation may act as a mechanism by which stress can induce depression. A variety of stress paradigms are used preclinically to study the onset and development of depressive-like behaviors (for review, see Stepanichev et al., 2014). Furthermore, it is thought that chronic stressors such as social defeat or chronic mild stress may reflect the clinical condition more closely. More specifically, studies assessing the impact of chronic mild stress in rodents have revealed that prolonged exposure to mild stressors induces anhedonic behaviors, evidenced by a reduced preference for sucrose solutions (Grippo et al., 2005). In accordance with this, naïve mice subjected to a variety of stressors for a 5-week period also displayed depressive-like behavior and decreased sucrose preference in addition to reduced social exploration concomitant with brain inflammation, as evidenced by elevated hippocampal IL-1 levels (Goshen et al., 2008). However, mice with an IL-1 receptor type 1 deletion subjected to a similar chronic mild stress regime did not display the depressive-like behavior observed in naïve mice, suggesting that elevated CNS IL-1 levels, as a consequence of prolonged stress, are responsible for the induction of depressive symptomology via adrenocortical activation and the suppression of neurogenesis (Goshen et al., 2008).

Early-life stress also has a crucial impact upon the development of neurobiological systems implicated in stress and mood responses, thereby increasing one’s vulnerability to stress and hence the risk of developing depression later in life, especially in response to secondary stress (Hammen et al., 2000; Mazure et al., 2000; Chapman et al., 2004; Nemeroff, 2004; Heim and Binder, 2012; Cattaneo et al., 2015). Preclinical studies assessing the effects of maternal separation in adult offspring have reported an altered stress response system in association with a proinflammatory phenotype (O’Mahony et al., 2009). Additionally, recent findings by Slusarczyk et al. (2015) highlight the detrimental effect of prenatal stress on microglial activity in adult offspring, evidenced by an elevated presence of inflammatory and microglial activation markers in the hippocampus and frontal cortex, in association with increased anhedonic and depressive-like behaviors. Similarly, clinical studies reveal a causal relationship between prenatal maternal stress and a dysregulated inflammatory profile in adolescent children (Veru et al., 2015). Furthermore, Frodl et al. (2012) report a positive association between childhood maltreatment and the general inflammatory marker and acute phase protein, CRP, in a depressed cohort. In accordance with this, depressed patients with a history of childhood trauma are often characterized by an elevated inflammatory signature in association with glucocorticoid resistance (GR) and altered brain derived neurotrophic factor (BDNF) concentrations, both thought to be as a consequence of the persistent, chronic, low-grade inflammatory phenotype (Heim et al., 2008; Mondelli et al., 2011).

Preclinical studies indicate that psychological stress increases gut permeability, thereby enabling gut flora to access the systemic system (Bowe and Logan, 2011). In line with this observation, Maes et al. (2008) have reported the presence of antibodies against endotoxin from a number of commensal bacteria in plasma from depressed patients. Consequently, it is possible that the potent innate immune stimulus and bacterial endotoxin, lipopolysaccharide (LPS), found on the outer cell wall of gram negative bacteria could stimulate a systemic, low-grade inflammatory response in depressed patients, although this remains to be fully elucidated.

The mechanistic driving force underpinning stress-induced immune activation involves the synergistic effects of the HPA axis, the sympatho-adrenal-medullary (SAM) axis, and the parasympathetic nervous system (PNS) (for review, see Miller et al., 2009). Under stress, in vitro and in vivo studies have shown that adrenergic receptor activation by noradrenaline culminates in the activation of inflammatory signalling pathways and the production of inflammatory cytokines (Miller et al., 2009). Catecholamines acting through alpha and beta adrenoceptors have been shown to have immunoregulating properties (Johnson et al., 2005). Stimulation of beta2 adrenoceptors on immune cells leads to the production of cAMP, which promotes the production of cytokines that can suppress cell-mediated immune responses (Suberville et al., 1996; Platzer et al., 2000). For instance, activated monocytes and dendritic cells exposed to noradrenaline have reduced IL-12 concentrations in parallel with increased production of the antiinflammatory cytokine IL-10 (Elenkov et al., 1996). Sustained activation of the SAM axis with chronic stress results in substantial increases in circulating catecholamine concentrations, and this can lead to adaptive changes in the expression of beta2-adrenoceptors. For example, it has been demonstrated that continuous exposure to catecholamines leads to a reduced receptor response by a process of receptor phosphorylation, internalization, and downregulation (Bawa-Khalfe et al., 2007). Therefore, in a similar fashion to GRs, stress-induced reduction of beta2 adrenoceptors could be involved in a diminished negative feedback response to catecholamines on immune cells. Conversely, others have reported an increase in catecholamine reactivity in mice subjected to chronic mild stress (Edgar et al., 2002, 2003). Lymphocytes from mice subjected to chronic mild stress have an increased response to catecholamine-mediated inhibition or enhancement of proliferation in T and B cells coupled with an increase in beta2 adrenoceptor density and responsivity. In addition, the PNS may also play a role in the regulation of inflammation. Activation of efferent vagus nerve fibers, acetylcholine release, and subsequent alpha7 nicotinic receptor activation on immune cells can inhibit cytokine responses to endotoxin in laboratory animals (Pavlov and Tracey, 2005; for review, see Miller et al., 2009). Increased muscarinic receptor expression has been reported in T and B cells isolated from mice exposed to chronic stress (Edgar et al., 2002). Thus, long-term adaptive responses are also evident in the PNS in response to stress, which may also impact on immune function.

## Immunological Activation Induces “Stress-Like” Behaviors

Further to and in corroboration with the above-mentioned findings, numerous studies investigating the impact of immunological insult in naïve rodents indicate that immunological activation induces stress-like behaviors. IL-1β has a critical role to play in the manifestation of sickness behavior, a strategic physiological response induced by the immune system in an effort to conserve energy in order to fight infection (Hart, 1988; Dantzer, 2006). Symptoms of sickness behavior include fever, malaise, loss of appetite, and fatigue in association with an activated stress response system (Konsman et al., 2002). Preclinical evidence suggests that the actions of IL-1β in the brain have a pivotal role to play in the sickness response. This is supported by the manifestation of symptomology and sickness behavior following an immune challenge with IL-1β or the bacterial endotoxin LPS, which abates in the presence of the IL-1 receptor antagonist (IL-1Ra) (for review, see Dantzer, 2006). However, prolonged activation of IL-1β in association with IL-6 and TNF-α appears to result in a maladaptive sickness response and the subsequent manifestation of depressive symptoms (Dantzer et al., 2008).

As reviewed by Gadek-Michalska et al. (2013), chronic inflammation also has a negative impact on central HPA axis function. Preclinical evidence suggests that exposure to endotoxin in early life has detrimental consequences on normal HPA axis functioning, evidenced by increased corticosterone concentrations in combination with stress-mediated alterations in lymphocyte proliferative abilities (Shanks et al., 2000). Further to this, recent findings indicate that early-life inflammatory challenges in naive mice are associated with depressive-like behavior in adulthood in association with decreased prefrontal cortex GR phosphorylation in the absence of altered corticosterone levels (Dinel et al., 2014). Moreover, early-life exposure to inflammatory stimuli appears to be a vulnerability factor, as reexposure to endotoxin later in life resulted in memory impairment and altered neurogenesis (Dinel et al., 2014).

Furthermore, others have shown that IL-1β and to a lesser extent IL-6, TNF-α, and IL-2 have a role to play in the modulation of neurotransmitters (Dunn, 1992; Palazzolo and Quadri, 1992; Song et al., 1999; Zhu et al., 2006). Under stress, dysregulation of serotonergic neurotransmission in particular may be, in part, responsible for the alterations in mood, emotion, and cognitive processing that are major characteristics of a depressive episode. Reichenberg et al. (2001) have shown that healthy male volunteers treated with low-dose endotoxin display increased circulating concentrations of IL-1Ra, IL-6, and TNF-α, which are positively correlated with emotional and cognitive disturbances evidenced by depressed mood, memory impairments, and anxiety.

Acute activation of the inflammatory response system (IRS) with an adaptive component is an essential host defence mechanism against stress and infection. Under normal physiological conditions this process is self-limiting with a distinct termination. However, failure of the IRS to resolve results in the subsequent development of a maladaptive, chronic, low-grade inflammatory process with detrimental consequences. Evidence of a low-grade inflammatory phenotype is apparent in numerous disorders such as cardiovascular diseases, obesity, type 2 diabetes, asthma, and psychiatric disorders such as major depression. It is not clear what instigates this maladaptive process, although Medzhitov (2008) suggests that the emergence of chronic inflammation may be a consequence of impaired homeostasis and the dysfunction of physiological systems not necessarily associated with host defence or tissue regeneration, which are commonly the principal initiators of an inflammatory response.

## Evidence for Activation of the Immune System In Major Depression

The monocyte-T-lymphocyte theory of depression, devised by Smith and Maes (Smith, 1991; Maes et al., 1995b) in the early 1990s, proposes that increased proinflammatory cytokine secretion in the form of IL-1β, TNF-α, and IFN-γ is responsible for the initiation and maintenance of a depressive episode. While the earliest studies by Kronfol et al. (1983) and Schleifer et al. (1984), investigating alterations in immune function in stressed and depressed individuals, reported a decreased T-cell proliferative response upon mitogen stimulation, much of the research to date has largely focused on the involvement of the innate immune response with numerous reports highlighting an association between major depression and activation of the innate immune response (Dantzer et al., 2008; Miller et al., 2009; Anisman, 2011; Leonard and Maes, 2012). In particular, evidence suggests that depression is associated with increased circulating concentrations of proinflammatory cytokines such as IL-1β, IL-6, TNF-α, and IFN-γ in association with elevations in chemokines and the acute phase protein, CRP (Maes et al., 1995a; Lanquillon et al., 2000; Cizza et al., 2008; Simon et al., 2008; Diniz et al., 2010; Hughes et al., 2012). Additionally, in the case of IL-6, IL-1, TNF-α, the soluble IL-2 receptor (sIL-2R), and CRP, these results have been supported by meta-analyses (Dowlati et al., 2010; Haroon et al., 2012; Liu et al., 2012; Haapakoski et al., 2015).

Further to this, it has also been shown that cytokine immunotherapy in the form of IL-2 and IFN-α for the treatment of hepatitis C (Hep C) and certain types of cancer, such as malignant melanoma, can induce depression in 30% to 50% of these patients who are otherwise psychiatrically normal (Capuron et al., 2000; Bonaccorso et al., 2001; Capuron et al., 2001). Additionally, increased concentrations of monocytic cytokines such as IL-6, in association with indicators of cell-mediated immune activation and T-cell subset cytokine production, have been highly associated with the onset of depressive symptoms following IFN-α treatment (Bonaccorso et al., 2001; Wichers et al., 2007). Significant symptom overlap between idiopathic and cytokine-induced depression has been observed (Capuron et al., 2009). Moreover, the development and progression of the cytokine-induced depressive symptoms can be inhibited with antidepressant treatment, suggesting that the therapeutic efficacy of antidepressants may be related to their immunomodulatory properties (Musselman et al., 2001; Raison et al., 2005).

In accordance with this, numerous studies have reported on the antiinflammatory properties of antidepressants (for review, see Kenis and Maes, 2002). Specifically, Kubera et al. (2001) suggest that antidepressants may exert their effects via immunoregulatory mechanisms evidenced by elevated IL-10 concentrations and a suppressed IFN-γ/IL-10 production ratio in stimulated whole blood from severely depressed patients treated with antidepressants in vitro. Others have shown reduced whole blood TNF-α concentrations in patients who responded to a 6-week course of amitriptyline (Lanquillon et al., 2000), while Seidel et al. (1995) reported a normalization of the elevated IFN-γ and sIL-2R concentrations in a patient cohort following antidepressant treatment. Increased TNF-α plasma concentrations were reduced following a course of electroconvulsive therapy (Hestad et al., 2003), while others have shown that treatment with selective serotonin reuptake inhibitors can reduce circulating CRP concentrations in the absence of therapeutic efficacy (O’Brien et al., 2006). Additionally, antiinflammatory agents have been shown to enhance the efficacy of antidepressant treatment with findings by Muller et al. (2006) showing an enhanced antidepressant efficacy of the noradrenaline reuptake inhibitor reboxetine when given in combination with the cyclooxygenase-2 inhibitor, celecoxib, a known inhibitor of prostaglandin E2 and proinflammatory cytokines, while Raison et al. (2013) have shown that depressed patients with higher baseline inflammatory markers respond to treatment with the TNF-α antagonist, infliximab. It is noteworthy however, that the meta-analysis by Hannestad et al. (2011), which assesses the effect of antidepressant treatment on serum cytokines levels of IL-1, IL-6, and TNF-α in 22 independent studies, report that while antidepressant treatment appears to reduce levels of IL-1 and possibly IL-6, TNF-α levels were not reduced in accordance with reduced depressive symptomology.

An increased prevalence of depression has also been observed in association with autoimmune disorders with up to 50% of multiple sclerosis patients developing clinical depression (Feinstein, 2011). Additionally, the chronic inflammatory disorder rheumatoid arthritis is also highly associated with clinical depression symptoms with up to 42% of patients reporting comorbid depression (Bruce, 2008). Therefore, the presence of an autoimmune disease appears to put people at a risk 3 times greater than that posed to the general population.

Elevated T-cell-stimulated inflammatory cytokine production prior to experiencing a stressful life event such as military deployment has recently been shown to be a risk factor for the development of depression (van Zuiden et al., 2011). This finding represents the emergence of a new body of literature, once again addressing the involvement of the adaptive immune response in the pathogenesis of depression.

## Adaptive Immune System Activation in Depression

The innate and adaptive immune response collaborate in a bidirectional manner to maintain a homeostatic balance (Reiner, 2007). Increased IL-2 receptor expression and secretion upon T-cell activation promotes T-cell proliferation and differentiation (Medzhitov and Janeway, 1997). Additionally, the production of either proinflammatory (IFN-γ and IL-12) or antiinflammatory (IL-4 and IL-10) cytokines also has a key role to play in T-cell polarization (Medzhitov and Janeway, 1997). The complex adaptive cellular immune network and differentiation of T-cells into proinflammatory (Th-1 and Th-17), antiinflammatory (Th-2), or regulatory (T-reg) results in direct and specific immune responses to various pathogens as well as offering protection against potentially harmful effector responses and autoimmunity.

Furthermore, T-cell activation results in the further production of various cytokines. More specifically, and in relation to the discussion in the following paragraph, the production of IFN-γ from Th-1 cells promotes further activation of monocytes and macrophages, resulting in the additional release of monocytic cytokines such as IL-1, macrophage inflammatory mediators such as neopterin, and interferon-inducible genes, thereby sustaining a proinflammatory phenotype (Zhou et al., 2009; Maes, 2011). The interaction between T-cells and macrophages via the production of a range of inflammatory cytokines is known as cell-mediated immune activation and functions to stimulate and recruit new macrophages, kill infected macrophages, and maintain the inflammatory response (Maes, 2011).

As reviewed by Miller (2010), during the last 10 years the research focus has shifted with a reemergence of reports in corroboration with early theories by Smith and Maes (Maes et al., 1995b) suggesting the involvement of the adaptive immune response and cell-mediated immune activation in the pathophysiology of major depression. In the early 1990s elevated macrophage-secreted neopterin levels, which represent increased IFN-γ-mediated macrophage activation, in association with increased circulating concentrations of the cell-mediated immune activation markers sIL-2R were observed in depressed and melancholic patients, suggesting an elevated presence of cell-mediated immune activation (Caruso et al., 1993; Maes, 1995). In support of this, findings by Celik et al. (2010) also suggest increased cell-mediated immune activity evidenced by elevated neopterin concentrations that are positively correlated with recurrent depression and an increased number of depressive episodes. Additionally, some reports suggest an imbalance in T-cell subset cytokine production with elevated levels of prototypical Th-1 cytokines, such as IFN-γ, while antiinflammatory IL-4 and TGF-β produced by Th-2 and Th-3 cells, respectively, have been found to be significantly lower in depressed cohorts (Myint et al., 2005; Sutcigil et al., 2007). However, it is important to note that the literature is varied in this regard (Pavon et al., 2006; Kim et al., 2007). In addition, others have reported elevations in both pro- and antiinflammatory cytokines (IFN-γ, IL-6, IL-7, IL-8, IL-10, IL-1Ra, and G-CSF) in depressed cohorts relative to controls that were then normalized following therapeutic intervention (Dahl et al., 2014) (Table 1). It is also noteworthy and apparent in Table 1 that some of the discrepancies in the literature arise as a consequence of a number of highly variable and influential factors. These include the wide variety of depressive subtypes investigated (endogenous, reactive, melancholic, or atypical), first episode vs recurrent or treatment-resistant depression, depression severity, comorbid disorders, the presentation of a diverse range of symptoms (affective or somatic), medical history, medication status, the immunological profile under assessment, the number of participants included, and the methods employed in the study.

**Table 1.** Summary of the Dysregulation of Th-1/Th-2 Cytokines in Depressive Disorders

Participants	Sample Size	Diagnostic Test Employed	Study Design	Medication Status	Inflammatory Mediators Investigated	Source	Study Methods Employed	Baseline Inflammatory Profile	Inflammatory Profile Post Treatment	Reference
MDD and healthy controls	-30 depressed patients (9 first episode, 12 second episode and 9 third episode) -26 controls	SCID-1 and HAM-D (17 item)	Controls vs depressed	Patients were free of psychoactive medication for 6 weeks prior to the study	Neopterin	Serum	-HPLC	-Increased neopterin levels in patients with recurrent depression relative to controls and first onset patients -Neopterin levels increased in accordance with the number of previous episodes	N/A	(Celik et al., 2010)
Mixed depressive subtypes and nondepressed controls	-238 cases with depressive symptoms (Inc. major, minor depression and dysthymia) -357 non- depressed controls	CES-D	Control vs depressed		Neopterin	Plasma	-HPLC	-No difference in neopterin levels between depressed subjects and controls -No association between neopterin levels and depressive symptoms	N/A	(Tiemeier et al., 2006)
MDD and healthy controls	-40 depressed patients (22 completed the 8 week study) -80 controls	HAM-D	-Controls vs depressed -Pre- and post- antidepressant treatment	Patients were medication naïve (first onset) or medication free for 4 months prior to the study	IFN-γ, IL4 and TGFβ1	Plasma	-8 weeks course of antidepressants -ELISA	-Increased IFN-γ/IL-4 and IFNγ/TGF-β1 ratio relative to controls -Negative correlation between TGF-β1 and HAM-D scores	-Decreased IFN-γ / IL-4 ratio post- treatment relative to pre-treatment -Increased TGF-β1 post-treatment relative to pre- treatment levels	(Myint et al., 2005)
MDD and healthy controls	-23 depressed patients (first episode) -25 controls	HAM-D	-Controls vs depressed -Pre- and post- antidepressant treatment	Patients were medication free for 6 weeks prior to the study	IL-2, IL-4, IL-12, TNF-α, TGF-β1 and MCP-1	Serum	-8 week course of sertraline treatment -ELISA	-Increased levels of IL-2, IL-12, TNF-α and MCP-1 relative to controls -Lower levels of IL-4 and TGF-β relative to controls	-IL-12 levels decreased and IL-4 and TGF-β levels increased post treatment relative to pre-treatment levels	(Sutcigil et al., 2007)
MDD and healthy controls	-32 depressed patients -63 controls	HAM-D	Controls vs depressed -Pre- and post- antidepressant treatment		IL-6, TNF-α, IFN-γ, IL-2, IL-4, TGF-β1	Whole blood	-6 weeks course of antidepressants *-In-vitro* stimulation with phyto- hemagglutinin (4μg/ ml) and LPS (20μg/ ml) for 48hrs -ELISA	-Increased production of IL-6, TNF-α, TGF-β1 and IFN-γ/IL-4 ratio relative to controls -Decreased production of IFN-γ, IL-4 and IL-2 relative to controls	-IL-6 and TGF- β1 levels were decreased post treatment relative to pre-treatment levels	(Kim et al., 2007)
MDD (with comorbid anxiety in some cases) and healthy controls	-33 depressed patients (80% first episode, 20% recurrent depression) -33 controls	HAM-D (17 item)	Control vs depressed	Patients were medication free for 3 weeks prior to the study	TNF-α, IL-6, IL-1β, IL-2, IFN-γ, IL-4 and IL-13	Serum	-ELISA	-Increased levels of TNF-α, IL-4 and IL-13 relative to controls -Decreased levels of IL-2 and IFN-γ	N/A	(Pavon et al., 2006)
MDD patients and healthy controls	-50 depressed patients (76% had melancholic depression at baseline; 43 completed the follow-up assessment) -34 controls	IDS scale	Controls vs depressed -Pre- and post- antidepressant treatment	Patients were medication free for 3 weeks prior to the study	IL-1β, IL-1Ra, IL-5, IL-6, IL- 7, IL-8, IL-10 and G-CSF	Plasma	-12 weeks course of antidepressants (treatment as usual) - Bio-Plex Pro Human Cytokine Group I with the Luminex 100	-Increased levels of IL-1, IL-1Ra, IFN-γ, IL-5, IL-6, IL-7, IL-8, IL-10 and G-CSF relative to controls	-Decreased levels of IL-1Ra, IFN-γ, IL-6, IL-7, IL-8, IL-10 and G-CSF post- treatment relative to pre-treatment levels and did not differ relative to controls -Normalization of cytokine levels only observed in treatment responders	(Dahl et al., 2014)

Abbreviations: CES-D, The Centre for Epidemiologic Studies Depression Scale; ELISA, enzyme-linked immunosorbent assay; G-CSF, granulocyte-colony stimulating factor; HAM-D, The Hamilton Rating Scale for Depression; HPLC, High Performance Liquid Chromatography; IDS, Inventory of Depressive Symptomology scale; IFN, interferon; IL, interleukin; MCP-1, monocyte chemoattractant protein-1; MDD, Major Depressive Disorder; SCID-1, The Structured Clinical Interview for Depression; TGF, transforming growth factor; TNF, tumor necrosis factor.

There are many inconsistencies in the literature largely owing to the fact the major depression is a highly heterogeneous disorder and therefore discrepancies in the immunological profile arise as a consequence of a number of highly variable and influential factors. ‘These include the wide variety of depressive subtypes investigated (endogenous, reactive, melancholic, or atypical), first episode vs recurrent or treatment-resistant depression, depression severity, co-morbid disorders, the presentation of a diverse range of symptoms (affective or somatic), medical history, medication status, the immunological profile under assessment, the number of participants included, and the methods employed in the study’. These factors are highlighted in the text and are further illustrated in the table which summarizes the variable nature of studies undertaken and the results obtained, particularly in relation to the adaptive immune response and the imbalance/dysregulation of T-cell subset cytokines in depression.

It has also been suggested that the balance between inflammatory Th-17 cells and immunoregulatory T-reg cells may be disturbed in depressed patients in favor of an increased proinflammatory Th-17 cell profile (Haroon et al., 2012). While there is little direct evidence to support or refute this, it may be conceivable in light of recent preclinical findings by Beurel et al. (2013) that directly implicate stress-induced elevations of brain Th-17 cells in the promotion of depressive-like behaviors in mice, which are then attenuated following the targeted inhibition of Th-17 cell production and function. In addition, Th-17 cells are highly implicated in the pathogenesis of inflammatory disorders such as rheumatoid arthritis, multiple sclerosis, and inflammatory bowel disease, often presenting comorbid with depression (Wilson et al., 2007; Tesmer et al., 2008). On the other hand, with regard to T-reg cells and in further support of this theory, Li et al. (2010) reported a decreased expression of CD4^+^CD25^+^ T-reg cells in unmedicated, first-episode, melancholic major depressed patients relative to healthy age- and sex-matched control subjects. Moreover, Himmerich et al. (2010) reported that, in accordance with a decreased expression of IL-1 and IL-6 during antidepressant therapy, expression of T-reg cells was increased in patients who had been suffering from a mild depressive episode, while a significantly larger increase in CD4CD25hi cells posttreatment was evident in the more severely depressed patients. Seemingly contrary to the above-mentioned findings, recent studies investigating T-cell and monocytic inflammatory systems in bipolar and schizophrenia patients reported an elevated presence of T-reg cells in association with a monocytic inflammatory signature (Drexhage et al., 2011a, 2011b). Additionally, Ronaldson et al. (2015) also reported an elevated T-reg cell presence in association with blunted cortisol and increased IL-6 levels in a healthy cohort of volunteers following acute stress. The elevated T-reg cell presence was also found to be correlated with worse physical and mental health status and higher levels of depressive symptomology (Ronaldson et al., 2015). Conversely, Drexhage et al. (2011a, 2001b) suggest that a higher T-reg cell presence in psychiatric patients at admission was associated with better clinical outcome at discharge.

This is of interest in the context of stress and depression, with alterations in immune proliferative responses frequently observed in depressed and stressed cohorts. Furthermore, regulatory T-cells can also be induced under circumstances of low tryptophan availability as a consequence of increased indoleamine 2,3 dioxygenase (IDO) activity (Fallarino et al., 2006), which is thought to be key in linking inflammation and kynurenine pathway activation in depression as discussed below. Consequently, the elevated or prolonged presence of T-reg cells may represent a vulnerability factor or early marker for major depression.

It may seem, however, that the earliest reports investigating the association between inflammation and depression are in contrast to a more active T-cell role, with the suggestion that stress and severe depression negatively affect T-cell function with reduced responses upon mitogen stimulation (Zorrilla et al., 2001; Irwin and Miller, 2007; Miller, 2010). While an immunosuppressed T-cell response in the face of immune challenge may appear in contrast with the proinflammatory signature that severely depressed patients are often characterized by, it is possible that inflammation itself (as maybe the case with T-reg cells) and a dysregulated stress response may also have a part to play in the diminished T-cell responses in major depression (Miller, 2010). This too is reasonable given the findings from numerous studies investigating the disruptive impact of chronic TNF-α exposure on T-cell proliferation, cytokine production, and apoptosis and its disruptive effects on T-cell receptor signalling and NFκB activation, culminating in nonresponsive T-cells (Cope et al., 1994, 1997; Lee et al., 2008; Nie et al., 2013). Furthermore, chronic inflammation is also thought to impair GR expression and function in addition to promoting a steroid refractory phenotype via the expression of the inactive beta isoform of the GR (Goleva et al., 2002; Wang et al., 2004; Schewitz et al., 2009). As a consequence, T-cells may no longer be capable of responding to vital neuroendocrine trafficking signals and the mobilization of T-cells to the brain, where, in times of stress, they impart neuroprotective effects (Miller, 2010). As such, alterations in T-cell function, trafficking, and immune regulation as a consequence of persistent inflammation and GR may have detrimental consequences on CNS responses (Miller, 2010).

## Biological Mechanisms Implicated in Depression Associated With Inflammatory Markers

### The Kynurenine Pathway

In recent years, theories linking the serotonergic and cytokine hypotheses of depression have emerged (Maes et al., 2011; Leonard and Maes, 2012). In support of this, preclinical reports studying animal models of depression have highlighted a possible role for kynurenine pathway activation (Figure 3) in the biological basis of depression (O’Connor et al., 2009a, 2009b). Specifically, O’Connor et al. (2009b) report that blockade of IDO activation in naïve mice following peripheral administration of the bacterial endotoxin LPS prevents the development of depressive-like behavior. Additionally, kynurenine administration to mice dose-dependently induces depressive-like behavior as assessed using the forced swim and tail suspension tests (O’Connor et al., 2009b). Moreover, while Bacille Calmette-Guérin induces inflammation, IDO activity and subsequent depressive-like behavior in wild-type mice, IDO-knockout mice inoculated with Bacille Calmette-Guérin display an increased inflammatory profile in the absence of IDO activity and depressive-like behavior, thereby suggesting that IDO has a central role to play in the onset of depressive symptomology (O’Connor et al., 2009a).

Further evidence in support of increased kynurenine pathway activation and subsequent tryptophan depletion in depression stems largely from the study of cytokine-induced depression, which occurs in 30% to 50% of medically ill patients being treated with IL-2 or IFN-α for cancer and Hep C (Musselman et al., 2001; Capuron et al., 2002; Capuron and Miller, 2004). However, it has also been shown that cytokine-induced depression severity is dose dependent; hence, the larger the dose of IFN-α, the greater the depression severity (Schaefer et al., 2002). Therefore, while this is strong evidence in support of the proposed involvement of the kynurenine pathway in depression, idiopathic depression is largely characterized by a low-grade inflammatory phenotype, and it is questionable if the inflammatory profile observed in medically healthy depressed patients is robust enough to induce an elevated kynurenine pathway activation and subsequent tryptophan depletion.

In accordance with this, to date, literature directly associating increased kynurenine pathway activation and idiopathic major depression is scarce. A cross-sectional study by Myint et al. (2007) reports an increased KYN/TRP ratio in the absence of changes in kynurenine and tryptophan alone and decreased neuroprotective kynurenic acid (KYNA) concentrations in the depressed patients relative to controls, while Gabbay et al. (2010) also report an increased KYN/TRP ratio in adolescents with melancholic depression. However, as there was no change in kynurenine, the increase in the KYN/TRP ratio appears to be solely as a consequence of the significant decrease in tryptophan. Yet, increased measures of kynurenine pathway activation were associated with depression severity in the melancholic depressed patients (Gabbay et al., 2010). One study does report an elevated serum concentration of kynurenine and depleted tryptophan and 5HIAA availability in a mixed cohort of bipolar and depressed patients relative to healthy controls (Myint et al., 2013). However, recent reports by Dahl and colleagues (Dahl et al., 2014, 2015) suggest that despite the presence of a dysregulated inflammatory profile in a cohort of unmediated depressed patients at baseline, there was no difference in kynurenine metabolite plasma markers or tryptophan concentrations relative to healthy controls at baseline. Furthermore, in accordance with a recent report by Hughes et al. (2012) of decreased tryptophan availability in a cohort of depressed patients, Maes and Colleagues (Maes et al., 2011; Maes and Rief, 2012) report that while depression and somatic disorders are both characterized by decreased tryptophan concentrations, there was no evidence in support of increased IDO activity in depression.

It has also been suggested that depression is not associated with a decrease in tryptophan per se but rather the downstream catabolites of the kynurenine pathway. This theory is supported by findings by Raison et al. (2010), where they showed that while tryptophan concentrations are depleted in the periphery of Hep C patients being treated with IFN-α, examination of CSF tryptophan concentrations revealed that the concentration of central tryptophan was normal. However, an increased kynurenine concentration observed in both the periphery and CNS and the associated elevations in CSF quinolinic acid (QUIN) and KYNA were found to correlate with the elevated inflammatory profile and depressive symptomology. Consequently, it appears that while tryptophan is maintained in the CNS, a decreased concentration of tryptophan in the periphery may be reflecting changes in kynurenine metabolites in the CNS. Further to this, it has been suggested that it is the kynurenines and perhaps an imbalance between the neurotoxic (QUIN) and neuro-protective (KYNA) catabolites that are responsible for the induction of depressive symptomology (Maes et al., 2011). This is further supported by recent findings showing that while kynurenine metabolite concentrations were unaltered in independent cohorts of depressed and remitted patients, the ratio of KYNA to QUIN was decreased in both patient cohorts relative to controls (Savitz et al., 2015a, 2015b). Furthermore, this reduced KYNA to QUIN ratio was inversely correlated with the number of depressive episodes in the remitted group, whereas in the depressed cohort, while no significant correlation was observed with depression severity, the reduced ratio of KYNA/QUIN was significantly correlated with anhedonia (Savitz et al., 2015b).

Furthermore, a study by Capuron et al. (2009) has shown that while there is considerable symptom overlap between cytokine-induced depression and idiopathic major depression, patients with IFN-α-induced depression display greater somatic symptom severity in the form of psychomotor retardation and weight loss relative to idiopathic depressed patients. This is very interesting in light of the report by Maes and Rief (2012) that concludes that kynurenine pathway activation is more pronounced in comorbid somatisation and depression compared with depression alone and suggest that changes in kynurenine pathway metabolites (tryptophan catabolites) may be more closely associated with somatic symptomology rather than depression per se, although this is in contrast with the above mentioned finding, showing an association between anhedonia and altered kynurenine pathway metabolites (Savitz et al., 2015a, 2015b).

## GR

While prolonged elevations in cortisol may be a consequence of chronic stress, alterations in GR function and sensitivity are also commonly reported in depressed individuals. Evidence in support of decreased glucocorticoid sensitivity, commonly referred to as GR, in depressed patients arises from studies assessing GR response to the synthetic glucocorticoid dexamethasone and the dexamethasone-CRH stimulation test with studies repeatedly reporting impaired glucocorticoid responsiveness and nonsuppression of cortisol secretion, which has been shown to correlate with depression severity and age (Carroll et al., 1981; von Bardeleben and Holsboer, 1991; Holsboer, 2000; Pariante and Miller, 2001; Ising et al., 2007).

Further to this, it is thought that persistent inflammation observed in subgroups of depressed and stressed individuals may have a significant role to play in the onset and maintenance of GR, which in turn may fuel the chronic inflammatory phenotype in a feed forward cascade. In support of this theory, assessment of GR expression on HeLaS3 cells, following stimulation with the inflammatory cytokine TNF-α, revealed an increased expression of the inactive beta (β) isoform relative to the active alpha (α) isoform of the GR. The increased protein expression of GRβ, which is unable to stimulate glucocorticoid inducible genes, was also associated with the development of GR (Webster et al., 2001). Additionally, elevated expression of GRβ has been detected in patients with inflammatory conditions such as arthritis and asthma (Sousa et al., 2000; Chikanza, 2002). Further to this, an elevated mitogen stimulated IL-1β and IL-6 production was positively associated with nonsuppressed plasma cortisol concentrations following the dexamethasone suppression test in a cohort of depressed patients (Maes et al., 1993a, 1993b). In accordance with this, Pariante and colleagues (Pariante et al., 1999) observed that IL-1α reduced GR translocation and decreased dexamethasone-induced GR-mediated gene activity by nearly 50% in a mouse L929 fibroblast cell line, further implicating a role for inflammation in the dysregulation and hyperactivity of the HPA axis.

**Figure 2.**
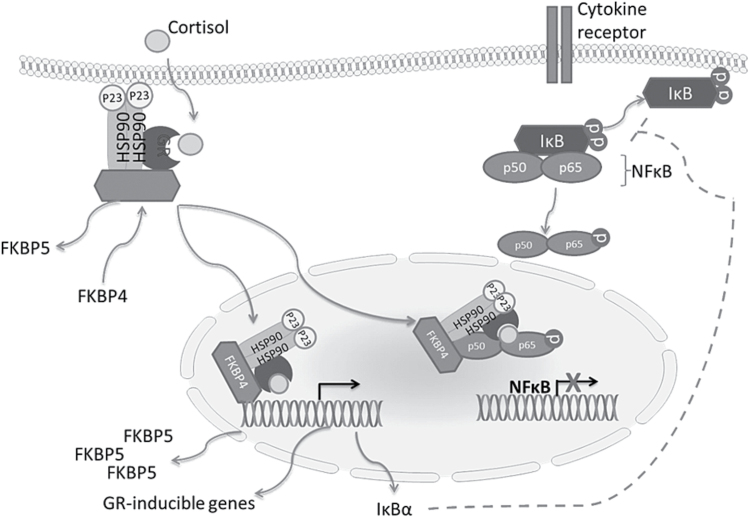
Glucocorticoid receptor (GR) signalling and suppression of inflammation under normal conditions. The GR is a ligand-activated transcription factor. Upon cortisol binding, it disassociates from its co-chaperone heat shock protein (hsp) complex in the cytosol and translocates to the nucleus. There it interrupts Nuclear Factor-Kappa B (NFκB) signalling through interactions with the NFκB regulator I kappa B alpha (IκB), thereby exerting antiinflammatory effects along with promoting the transcription of glucocorticoid-inducible genes such as glucocorticoid induced leucine zipper (GILZ) and serum and glucocorticoid regulated kinase 1 (SGK1) (Raison and Miller, 2003). The GR can also interact directly with the p65 subunit, preventing NFκB binding and the transcription of inflammatory cytokines. The mechanism of GR activation is tightly regulated by the co-chaperone hsp complex, which effectively controls the sensitivity of the receptor and consequently has been implicated in the pathogenesis of major depression. Specifically, under normal conditions, the hsp90 co-chaperone FK506 binding protein 51 (FKBP5) acts to negatively regulate the GR. When bound to GR, FKBP5 confers a low cortisol binding affinity on the receptor. However, upon cortisol binding and GR activation, FKBP5 is exchanged for FKBP4, permitting the translocation of the GR complex to the nucleus. This action, along with regulating and inducing gene transcription, also upregulates the expression of FKBP5, thereby decreasing the sensitivity of the receptor once again (Binder, 2009). (adapted from Smoak and Cidlowski, 2004).

Under normal physiological conditions, GR activation results in the production of antiinflammatory glucocorticoid-inducible genes and the activation of the hsp90 co-chaperone FK506 binding protein 51 (FKBP5), which acts as a negative regulator of GR activation (Figure 2). Interestingly, healthy controls carrying certain polymorphisms in the FKBP5 gene display an exaggerated increase in the transcriptional expression of FKBP5 upon GR activation in response to psychosocial stress, thereby decreasing the sensitivity of the receptor as evidenced by insufficient cortisol recovery and the persistent activation of the HPA axis (Ising et al., 2008). Additionally, Zimmermann et al. (2011) have shown that homozygotes of the minor allele of the FKBP5 gene are more vulnerable to the development of major depression in the wake of severe adverse stressful life events.

### Serotonin Transporter (SERT)

Further to this, the SERT, a transmembrane protein that removes serotonin from the synapse following its release (Benmansour et al., 1999), also appears to pose a risk for the development of a depressive episode following a stressful life event (Caspi et al., 2003). SERT may also represent a link between the inflammatory hypothesis and reduced serotonergic function in depression. In this regard, studies have demonstrated that inflammatory cytokines, including IL-1β, TNF-α, and IFN-α, increase SERT expression and serotonin reuptake in vitro (Zhu et al., 2006; Tsao et al., 2008). Additionally, a systemic inflammatory challenge with LPS increases SERT expression in the rodent brain (Zhu et al., 2010). A sustained increase in CNS transcriptional expression of IFN-α and SERT in response to a single systemic challenge with the viral mimetic Poly IC was also found to have functional significance, evidenced by an associated decrease in extracellular serotonin concentrations quantified in the patient cohort (Tsao et al., 2006). Furthermore, elevated expression of the inflammatory cytokines IL-1β, TNF-α, IL-6, and IFN-γ on blood leukocytes from depressed patients was associated with an increased transcriptional expression of SERT. However, following chronic selective serotonin reuptake inhibitors treatment, decreased transcriptional expression levels of IFN-γ and SERT were observed (Tsao et al., 2006).

### Neurotrophins

Both stress and inflammation can disrupt the expression and function of BDNF (Schaaf et al., 1998; Barrientos et al., 2003). BDNF has a central role in neuronal proliferation, regeneration and survival, neurogenesis, and synaptic plasticity (Tapia-Arancibia et al., 2004). In particular, Barrientos et al. (2003) have shown that alterations in hippocampal-dependent processes may be a consequence of IL-1β mediated decreases in BDNF. In accordance with this, Karege et al. (2005) have shown decreased BDNF expression in suicide victim postmortem hippocampal and prefrontal cortex tissue, while others have repeatedly reported reductions in the circulating concentration of BDNF in depressed patients (Karege et al., 2002; Cattaneo et al., 2010).

Moreover, while numerous studies have suggested a decrease in hippocampal neurogenesis associated with depression (Ekdahl et al., 2003; Kempermann et al., 2008; Boldrini et al., 2009) and furthermore that inflammation and specifically IL-1β has the capacity to mediate this (Goshen et al., 2008; Koo and Duman, 2008; Spulber et al., 2008; Kohman and Rhodes, 2013), the mechanism(s) of action remain to be elucidated. However, recent studies using human hippocampal progenitor cells have yielded novel in vitro findings, indicating that IL-1β medicated kynurenine pathway activation, with specific elevations in the expression of kynurenine monooxygenase and kynureninase, leading to the production of potentially neurotoxic metabolites, have detrimental effects on hippocampal neurogenesis (Zunszain et al., 2012).

### Synergy between Stress, Cytokines, and the HPA Axis

**Figure 3.**
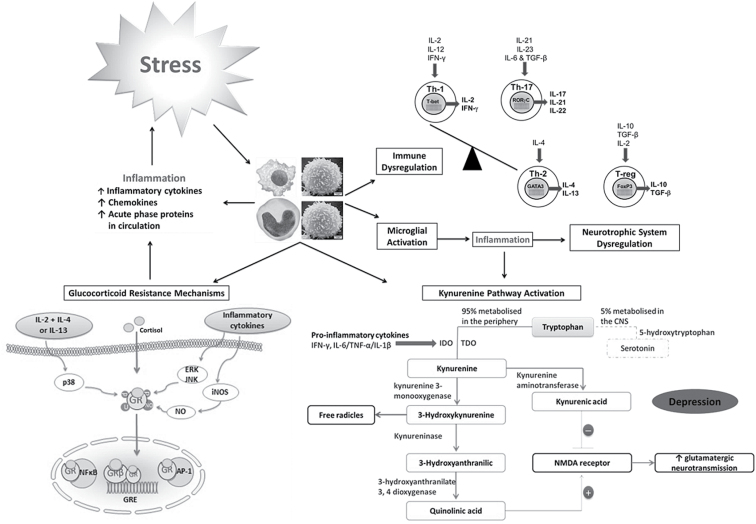
Summary of the mechanisms by which stress-related immune alterations may precipitate depression. Stress, psychological, physical, or combinational, is thought to be a major risk factor for depression. Chronic stress is thought to impact negatively on the inflammatory response system (IRS), potentially culminating in the manifestation of depressive symptoms. Specifically, a chronic inflammatory state, as a consequence of stress, may lead to activation and further dysregulation of both the innate and adaptive immune response, further promoting an inflammatory environment. The synthesis of serotonin in the CNS is dependent upon the availability of the essential dietary amino acid tryptophan (Russo et al., 2009). In this regard, the kynurenine pathway is the major metabolic pathway for tryptophan in the body, resulting in the production of kynurenine and many downstream metabolites. Indoleamine 2,3 dioxygenase (IDO) is the rate-limiting, tryptophan-degrading enzyme of the kynurenine pathway and is upregulated in response to immune activation. Specifically, IFN-γ is the most potent inducer of IDO activation. However, IFN-γ-independent mechanisms such as prostaglandin E2 or interleukin (IL)-6 in combination with TNF-α or IL-1β are also known inducers of IDO activity (Carlin et al., 1989; Fujigaki et al., 2006; Zunszain et al., 2012). In addition to IDO, activation of the hepatic tryptophan-degrading enzyme tryptophan, 2,3 dioxygenase (TDO) in response to psychological stress, glucocorticoids, or tryptophan itself, also results in kynurenine pathway activation in the liver (Moffett and Namboodiri, 2003). Therefore, IDO/TDO induction has been proposed as a mechanism by which stress and inflammation can precipitate depression via kynurenine pathway activation and tryptophan depletion. A chronic inflammatory state may alter serotonergic neurotransmission via the depletion of tryptophan and increased production of neurotoxic and excitotoxic mediators, which in association with chronic stress and inflammation itself, may have a negative impact on the neurotrophin system and BDNF concentrations in the brain. Stress-induced immune activation is also thought to contribute to the induction of HPA-axis hyperactivity and glucocorticoid resistance (GR), thereby inhibiting the potent antiinflammatory effects of cortisol, which, in turn, contributes further to a dysregulated inflammatory response. Stress-related immune dysregulation and subsequent alterations in monoaminergic neurotransmission, the stress response system and the neurotrophic system, alone or in combination, have a detrimental impact on normal brain functioning, potentially culminating in the manifestation of the behavioral and physiological alterations that currently characterise the depressive condition. GR mechanisms adapted from Barnes (2010, see for review). AP, Activator protein-1; GR, glucocorticoid receptor; GRE, glucocorticoid response elements; iNOS, inducible nitric oxide synthase; NO, nitric oxide; NFκB, nuclear factor kappa B; Th, T helper cells.

Taken together, the synergistic dysregulation and feed forward cascade of multiple biological systems appear to play a significant role in the onset of depressive symptomology (Anisman, 2009) (Figure 3). Stressful life events such as childhood trauma, genetics, and chronic activation of the IRS, alone or in combination, negatively impact on normal functioning and inhibitory control of the stress response system in association with altering the inhibitory feedback mechanisms of glucocorticoids on cytokine secretion, thereby inducing hypercortisolemia (or hypocortisolemia) and subsequently contributing further to the manifestation of a dysregulated inflammatory and neuroendocrine phenotype. These factors potentially culminate in the manifestation of the behavioral and physiological alterations that currently characterize the depressive condition. Furthermore, as discussed by Frodl and O’Keane (2013), preclinical evidence suggests that elevated cortisol concentrations may have an excitotoxic and neurotoxic impact on brain structures strongly associated with depressive symptomology. However, while hippocampal volumetric changes are evident in depressed patients (Frodl et al., 2012), further clinical assessments are required to decipher if putative neuronal alterations are a consequence of a dysfunctional stress response and elevated glucocorticoid and or cytokine concentrations.

### An Emerging Role for Microglia?

With increasing evidence of an inflammatory response in major depression, mechanisms of communication between the periphery and the brain, which include the circumventricular organs, active transport, and peripheral afferent nerve fibres (for review, see Connor and Leonard, 1998), provide a means by which large, peripherally produced cytokine molecules (17-51kD), unable to passively diffuse across the blood brain barrier due to their size and structure, can alter neuronal and glial cell function and behavior via cytokine receptor activation in the CNS, thereby potentially impacting on mood and the manifestation of depressive symptomology (Hopkins and Rothwell, 1995; Szelenyi, 2001). Despite this, little is known about the specific inflammatory state in the brain of MDD patients. However, as reviewed by Beumer et al. (2012), advances in brain imaging are opening up new avenues for investigation, with activated microglia found on brain scans of patients with psychiatric disorders. In accordance with this and of particular interest is the recent report by Setiawan et al. (2015), which presents evidence of brain inflammation and specifically microglial activation evidenced by the elevated presence of translocator protein density measured by distribution volume in a patient cohort during a major depressive episode. Furthermore, elevations in translocator protein density measured by distribution volume in the anterior cingulate cortex were robustly correlated with depression severity, suggesting that greater microglial activation in specific brain regions may result in the manifestation of specific depressive symptoms (Setiawan et al., 2015). Interestingly, however, contrary to the above-mentioned reports, preclinical findings by Kreisel et al. (2014) suggest that hippocampal microglial dysregulation, rather than activation, as a consequence of chronic stress may actually be responsible for the development of certain depressive symptoms. More specifically, Kreisel et al. (2014) show that while acute stress induces short-term microglial activation and proliferation, rodents subjected to 5 consecutive weeks of chronic unpredictable stress, in fact, exhibited downregulated microglial functional capacity and apoptosis in addition to depressive-like behavior. Moreover, subsequent immunological challenge, resulting in microglial activation and proliferation, ameliorated or reduced the anhedonic depressive-like behavior observed following CUS, thereby providing a causal link between microglial dysfunction and chronic stress-induced depression symptomatology (Kreisel et al., 2014). Consequently, microglial stimulators may prove to be beneficial in the treatment of some depressive subtypes (Kreisel et al., 2014). The mechanisms responsible for the activation of microglia in psychiatric disease remain elusive. A direct microbe-driven activation of microglia is possible (Kristensson, 2011), but apart from direct microbial/gDNA activation of microglia, inborn errors in the growth and differentiation of myeloid progenitor cells can make the cells vulnerable for hyper-stimulation. The identification of genetic risk factors and the role that genetics might have in these complex relationships is largely unexplored territory to date. As the technology to manipulate the genome has come of age, such questions can be adopted from the clinic and readily explored in animal models.

### Implications for Treatment

As the biological basis of depression still remains elusive, treatment strategies are not always effective and multiple trials often necessary to elicit a response. Consequently, in recent years, attention has focused on the identification of biological markers (biomarkers) for depression. Emerging evidence in the last 10 years highlights the importance of peripheral blood as a potential diagnostic tool for many diseases including psychiatric disorders (Tsuang et al., 2005) and especially as it may be used to assess the inflammatory signature in depressed patients. Its importance also arises from studies by Liew et al. (2006), who show that peripheral blood cells share approximately 80% of the transcriptome with 9 non-blood-related tissues; specifically, they found 81.9% of all genes expressed in the brain to be coexpressed in human blood cells. Given that circulating blood cells respond to the macro and micro changes occurring around them and come into close contact with brain regions such as the pituitary and hypothalamus, it has been proposed that peripheral blood cells may act as “surrogates for CNS expression” (Liew et al., 2006; Sullivan et al., 2006). Consequently, whole blood mRNA expression system may be thought of as a proxy measure for mRNA expression in brain (Hepgul et al., 2013). In addition isolated peripheral blood mononuclear cells provide insight into the profile of transcriptional expression in white blood cells that are easily obtained from living patients and enable links to be made between depression severity or clinical staging and the biological profile (Le-Niculescu et al., 2007). As such, changes in specific biological markers may be used to identify predisposing risk factors, distinguish depressive subtypes, predict the onset of depression, progression of the disorder or perhaps treatment response, thereby eradicating self-report systems and subjectivity and facilitating a more targeted and fast-acting approach to treatment (Bell, 2004; Le-Niculescu et al., 2007; Sunde, 2010; Schmidt et al., 2011; Lopresti et al., 2014). However, given the heterogeneity of depression and the large number of biological systems implicated in the aetiology of depressive disorders, to date, the diagnosis of depression is not etiologically or biologically derived (Mossner et al., 2007). However, recent findings by Rapaport et al. (2015) demonstrate the use of a beneficial and promising strategy, which involves using a combination of inflammatory makers to aid in the distinction and identification of responders to antidepressant treatment.

In addition, while numerous facets of the IRS have been assessed in depressed cohorts, there is a lack of in depth studies investigating the expression and function of chemokines and their receptors in depressive disorders. Given the reports associating chemokines with depressive disorders (Rajagopalan et al., 2001; Suarez et al., 2003), future studies should assess PAXgene chemokine expression as a potential biomarker for depression in addition to the assessment of circulating chemokine concentrations. Given their role in the mediation of immune cell trafficking to sites of inflammation or injury, chemokine-medicated immune cell trafficking to the brain in patients with major depression should be explored.

Furthermore, advances in neuroimaging and positron emission tomograph scanning in living patients also hold great promise for the identification of brain specific biomarkers. Given that depression is primarily a disorder of the CNS, assessment of microglial activation states and brain volumetric changes in depressed patients in association with peripheral immune markers may provide a more targeted and comprehensive approach in the search for biomarkers with an ultimate goal to develop personalized treatment strategies for patients with major depressive disorders (Doorduin et al., 2008; van Berckel et al., 2008; Frodl et al., 2012).The primary measure of depression severity is the 21-item Hamilton Rating Scale for Depression (HAM-D) 21 total score which is a routinely used, validated, and standardized assessment tool for major depression (Hamilton, 1960). However, the total score merely provides insight into the global depressive state (Shafer, 2006). Others have demonstrated the use of the HAM-D subscale approach in genetics research on major depressive disorder and in the assessment of antidepressant medication (Seretti et al., 1999; Yu et al., 2002). The majority of research to date has focused on comparisons between depressed patients and healthy controls and correlations with total depression severity; however, the unidimensional scales enable the connection of biological parameters to individual symptom clusters of major depressive disorder, thereby assessing the contribution of each parameter to the individual components of the disorder (Lee et al., 2011). Refined clusters of the HAM-D scale include core depression, anxiety, insomnia, and somatic symptoms (Shafer, 2006). Previous studies have demonstrated that external measures have different association profiles with individual symptom clusters within a test (Williams and Richardson, 1993; Barefoot et al., 2000). Further to this, Faries et al. (2000) reported that core subscales were more efficient at detecting change than the HAM-D total score. Consequently, the use of a more targeted, symptom-wise approach to decipher the underpinning pathophysiological mechanisms of individual symptoms may lead towards the development of specific biomarkers, thereby leading to the development of a more effective treatment strategy.

Strategies that attempt to address wide variation within animal studies by subdividing populations according to behavioral or physiological characteristics, for example, coping styles, will also help to unravel factors underlying susceptibility or vulnerability. Additional research employing stressors with a greater degree of ecological validity that challenge the natural defence and/or adaptive capacity of animals, for example, social stress or influence of early-life stress, serve to increase the face validity and relevance of existing models and practices. The concept that stress may predispose to a premature ageing of the immune response has been proposed (Glaser and Kiecolt-Glaser, 2005) and to explore this further, a greater emphasis on longitudinal investigations is required.

Patients with evidence of an activated IRS are considered less likely to respond to regular antidepressant therapy, and it is hypothesised that treatment resistant patients will respond to conventional treatment in combination with immunosuppressive therapy expected to dampen the inflammatory state of non-responders. Monocyte and microglial activation may be reversed by administration of immunosuppressive drugs, including nonsteroidal antiinflammatories (Muller, 2010; Khan et al., 2014), N-acetylcysteine (Dean et al., 2011), and minocycline (Levkovitz et al., 2010), although such treatments have not been systematically investigated in psychiatric disorders, with further potential for novel immunosuppressive interventions. Use of nonsteroidal antiinflammatories, N-acetylcysteine, minocycline, and novel immunomodulating drugs in animal models (reviewed by McGuinness and Harkin, 2015) could be examined for effectiveness in rectifying behavioral abnormalities. Models will enable in-depth studies on the molecular mechanism of immune mediated behavioral abnormalities and their correction by drug treatment.

There are new possibilities for treatments that target pathways by which the immune system influences the brain such as cytokines or growth factors and their downstream mediators or the activation of relevant CNS immune cell types (eg, microglia) to emerge. Results from trials to determine the efficacy of antiinflammatory drugs such as the use of anti-TNFα in patients with psoriasis (Kannan et al., 2013) with antidepressant potential or the adjunctive use of cyclooxygenase-2 inhibitors for treating depression (Muller et al., 2010) have been encouraging. Drugs acting on the HPA axis, GRs, and postreceptor signalling are being considered as new therapeutic possibilities with the potential to correct dysregulation of the HPA-immune axis in depression (for review, see Maric and Adzix, 2013). Drugs classified as anti-glucocorticoids (GR agonist, GR antagonists, dehydroepiandrosterone-DHEA, steroid synthesis inhibitors) are of interest for their capacity to correct glucocortiocoid-associated inflammation and/or neuronal damage in depression. Many of these trials are still at the early proof-of concept stage and likely to feature in future developments of new treatments for depression with associated dysregulated endrocrine-immune axis function. Moreover, treatments addressing the influence of stress and stress-induced activation of the SAM and HPA axes including behavioral interventions that address psychological and autonomic reactivity to stress such as psychotherapy, exercise, and meditation may have efficacy regarding both treatment and prevention of depression.

## Statement of Interest

None.
